# Editorial: Virtual reality for pain management

**DOI:** 10.3389/fpain.2023.1274613

**Published:** 2023-09-01

**Authors:** Aliaa Rehan Youssef, Mohammed Gumaa, Marcin Czub

**Affiliations:** ^1^ Department of Physical Therapy for Musculoskeletal Disorders and Surgery, Faculty of Physical Therapy, Cairo University, Giza, Egypt; ^2^ Department of Systems and Biomedical Engineering, Faculty of Engineering, Cairo University, Giza, Egypt; ^3^ TRUST Research Center, Cairo, Egypt; ^4^ Institute of Psychology, University of Wroclaw, Wroclaw, Poland

**Keywords:** pain, virtual reality, pain management, pain treatment, virtual reality

**Editorial on the Research Topic** Virtual reality for pain management

Virtual reality (VR) is an innovative rehabilitation technology that showed promising results in alleviating acute and chronic pain across diverse conditions and populations. Its effectiveness in pain management has been attributed to various mechansims. The four original and review articles in this Research Topic address the effectiveness of pain management in various population, the feasibility of this intervention as an adjunctive therapy, as well as the potential underlying mechanisms.

Wong et al. investigated the effect of a 30-minute VR intervention on labor pain perception and coping strategies in their qualitative observational propsective cohort study. This investigation was nested within a randomized control trial (RCT), aiming to explore the influence of VR on these aspects. The study cohort included twenty nulliparous, term women who experienced contractions at least every 5 min, displayed pain scores ranging from 4 to 7 on the Wong-Baker pain scale, and did not receive any pain medications.

The study's outcomes showcased a signficant increase in the perceived effectiveness of VR in enhancing self-efficacy for managing labor pain. Participants perceived the VR experience as a means of establishing a connection with their breath, inducing a sense of calmness, relaxation, and serving as a distraction from pain. Among the participants, 70% reported pain reduction, 60% observed a decrease in anxiety, and all participants expressed their willingess to endorse the VR experience for laboring patients.

It is important to recognize that while this study prioritizes the presentation of patients' experiences and opinion, rather than solely relying on quantitative changes in pain scores, the study's design focused on evaluating a single VR intervention and was conducted with a small sample size. These limitations need to be addressed in future studies.

Noble et al. in their narrative review explore the potential of combining spinal cord stimulation (SCS) and VR as complementary treatments for chronic pain management.

SCS is an established invasive procedure that stimulates the spinal cord to reduce pain signals, providing relief in conditions like failed back surgery syndrome and complex regional pain syndrome. VR, on the other hand, is a non-invasive therapy that uses immersive virtual environments to distract patients from pain stimuli and promote relaxation. Both SCS and VR have shown promise in managing chronic pain independently, but the idea of combining them to achieve a synergistic effect is a new area of interest.

The review discusses the limited existing research on combining VR with SCS, focusing on one study that utilized a custom VR application alongside SCS. Preliminary findings suggest potential benefits in reducing pain scores during and after VR combined with SCS, but further research is needed to validate these findings and explore the long-term effects.

The authors highlight the potential economic benefits of combining VR with SCS. While SCS has proven effective in the short term, its therapeutic effects may diminish over time. Concurrent VR-SCS is hypothesized to enhance the analgesic properties of both modalities and potentially lead to extended pain relief and reduced healthcare costs.

Nagpal et al. focused in their scoping review on investigating the management of chronic low back pain (CLBP) using VR interventions. The reviewed studies not only evaluated the safety and feasibility of VR but also explored its impact on various aspects of patients' lives. The findings revealed that VR was well-received by participants, with high levels of enjoyment and adherence to the VR training programs. Moreover, patients treated with VR showed a significant reduction in stress hormones, indicating its potential for stress relief in CLBP management. Additionally, the use of VR was associated with a noticeable improvement in the quality of life, as evidenced by the results of various QoL questionnaires utilized in the included studies.

The majority of the studies included in the review showed that VR training was more effective in reducing pain among patients with CLBP compared to various control groups. However, it was observed that this pain reduction did not significantly translate into improved functional performance for these patients. It is important to highlight that there was considerable heterogeneity in how functional performance was assessed across the reviewed studies.

As evident from various studies and reviews showing the significant pain reduction potential of VR in different populations, Hadjiat and Marchand conducted their review to explore the mechanisms through which VR mediates pain relief. The main proposed mechanism was distraction in which VR employs cognitive and affective endogenous modulation. The immersive environment created by VR gives individuals a sense of being in a different world, altering their response to painful stimuli and affecting their nociceptive neural signals. Moreover, interacting with the VR environment tends to divide a person's attention, reducing the available attention span to process pain signals from receptors.

Apart from the distraction mechanism, studies proposed several other mechanisms. Functional MRI studies revealed that VR could decrease activity in the cortical regions of the pain matrix. Another mechanism is VR pain attenuation, where VR affects various brain signaling components, including emotions, concentration, memory, and other senses such as visual inputs, altering pain perception. Additionally, some studies suggested conditioned pain modulation, where pain is reduced through a concomitant painful stimulus. However, there was significant heterogeneity among the included studies concerning the VR equipment and treatment duration. To gain a better understanding of the effectiveness and mechanism of action of VR, high-quality RCTs are needed.

In conclusion, the articles in this special collection underscore the significance of VR as a multifaceted tool in pain management, offering both immediate relief and potential long-term benefits. As the field of VR continues to evolve, high-quality RCTs and larger-scale studies will be essential to comprehensively establish its effectiveness, mechanisms, and optimal integration into clinical practice.

